# Modulation of Female Genital Tract-Derived Dendritic Cell Migration and Activation in Response to Inflammatory Cytokines and Toll-Like Receptor Agonists

**DOI:** 10.1371/journal.pone.0155668

**Published:** 2016-05-12

**Authors:** Muki S. Shey, Niren Maharaj, Derseree Archary, Sinaye Ngcapu, Nigel Garrett, Salim Abdool Karim, Jo-Ann S. Passmore

**Affiliations:** 1 Centre for the AIDS Programme of Research in South Africa (CAPRISA), University of KwaZulu-Natal, Durban, South Africa; 2 Department of Gynaecology and Obstetrics, Prince Mshiyeni Hospital, Durban, South Africa; 3 Department of Epidemiology, Mailman School of Public Health, Columbia University, New York, United States of America; 4 National Health Laboratory Services, Cape Town, South Africa; 5 Institute of Infectious Diseases and Molecular Medicine (IDM), Division of Medical Virology, University of Cape Town, Cape Town, South Africa; 6 Institute of Infectious Diseases and Molecular Medicine (IDM), Clinical Infectious Diseases Research Initiative (CIDRI), University of Cape Town, Cape Town, South Africa; Harvard Medical School, UNITED STATES

## Abstract

HIV transmission across the genital mucosa is a major mode of new HIV infections in women. The probability of infection may be influenced by several factors including recruitment and activation of HIV target cells, such as dendritic cells (DCs) and cytokine production, associated with genital inflammation. We evaluated the role of inflammatory cytokines and TLR signaling in migration and activation of genital tract DCs in the human cervical explant model. Hysterectomy tissues from 10 HIV-negative and 7 HIV-positive donor women were separated into ecto- and endocervical explants, and incubated with inflammatory cytokines (TNF-α, IL-1β, IL-8, MIP-1β) or agonists for TLR4 (LPS), TLR2/1 (PAM3) and TLR7/8 (R848). Migration (frequency) and activation (HLA-DR expression) of myeloid and plasmacytoid DCs and Langerhans cells were measured by flow cytometry. We observed that cytokines, LPS and PAM3 induced activation of migrating myeloid and plasmacytoid DCs. LPS induced a 3.6 fold lower levels of migration of plasmacytoid DCs from HIV-infected women compared with HIV-uninfected women (median activation indices of 2.932 vs 0.833). There was however a 4.5 fold increase in migration of Langerhans cells in HIV-infected compared with HIV-uninfected women in response to cytokines (median activation indices of 3.539 vs 0.77). Only TLR agonists induced migration and activation of DCs from endocervical explants. Hormonal contraception use was associated with an increase in activation of DC subsets in the endo and ectocervical explants. We conclude that inflammatory signals in the female genital tract induced DC migration and activation, with possible important implications for HIV susceptibility of cervical tissues.

## Background

Dendritic cells (DCs) are important immune sentinels and among the first cells to recognize invading pathogens, including HIV, and initiate an immune response [1]. This initial innate response may dictate the subsequent type of adaptive immune response mounted in response to an infection. Activation of an adaptive response by DCs is achieved through antigen presentation of pathogen-derived peptides to cells of the adaptive immune system, involving co-stimulatory molecules and cytokines [2]. The ability of DCs to produce cytokines and to up-regulate expression of HLA class I/II and co-stimulatory markers (including CD86, CD80 and CD40) makes them the primary and most efficient activators of adaptive immunity [3]. Several distinct DC subsets have been described, including plasmacytoid DCs (pDCs), myeloid DCs (mDCs), and Langerhans cells (LCs) which differ in phenotypic and functional properties [4, 5]. Myeloid DCs and pDCs are peripheral DCs, and can be recruited to tissues, while LCs cells are predominantly found in tissues such as mucosal epithelium of the cervicovaginal compartment [6]. Pattern-recognition receptors (PRRs) on DCs and other cells of the innate immune system identify pathogen-associated molecular patterns (PAMPs), derived from microbial pathogens or stressed cells. Of the 10 toll-like receptors (TLRs) in humans with either extracellular or intracellular localization, TLR1, 2, 4, 5, 6, and 10 are expressed at the cell surface and recognize microbial products that are not produced by the host, while TLR3, 7, 8, and 9 are located almost exclusively in endosomal compartments and are specialized in recognition of nucleic acids [7, 8]. DC subsets differentially express PRRs depending on their function and location [9]. The type of PRR initially triggered may determine the outcome of an innate immune response, as binding to different receptors may result in distinct immune outcomes [10].

The mucosa of the lower female genital tract provides the first line of defence against pathogen entry and mediates the initial host immune response [11–13]. In the female genital tract, it is thought that DCs capture HIV through a variety of receptors [14], and initiate an immune response to possibly kill the virus [5]. Apart from their role in activating T cells through antigen presentation, DCs are also thought to capture and directly transfer HIV to T cells in the genital mucosa by *trans*-infection, whereby they are not directly infected with HIV but transfer HIV to CD4 T cells for infection [15–18]. Whether they play a direct or indirect role in HIV infection, they are one of the first cell types recruited after infection, and cytokines produced by DCs are necessary for establishment of a productive systemic infection [19]. Thus, DCs may play a dual role of preventing and enhancing HIV transmission.

Pathogenic viral and bacterial infections in the lower genital tract are recognized by sentinel DCs and other immune cells, leading to inflammation [20, 21]. CD4^+^CCR5^+^ HIV target cells, including DCs, macrophages and CD4^+^ T cells present at the genital mucosa or recruited to this mucosa in response to pathogens, may also secrete inflammatory cytokines leading to the recruitment of more cells including DCs, monocytes/macrophages, neutrophils, NK and T cells [4, 22–26].

Several innate factors have been linked to the risk of HIV acquisition, including inflammatory cytokines and PRRs. Increased levels of several cytokines (including macrophage inflammatory protein [MIP]-1α, MIP-1β, interferon-γ inducible protein-10 [IP-10], interleukin [IL]-8, IL-1α, IL-1β, IL-6, IL-7, IL-10 and tumor necrosis factor-alpha [TNF-α]) have been associated with an increased risk of HIV acquisition in women participating in 1% TFV gel trial in South Africa [27]. This was thought to be due to an increase in recruitment of HIV target cells caused by these cytokines, potentially enhancing HIV infection. In addition, several TLRs and TLR pathway signaling molecules including TLR4, TLR7/8, TLR2 and myeloid-differentiation primary response protein 88 (MyD88) have been implicated in the risk for HIV transmission and HIV disease progression [28–31]. HIV gp120 is suggested to bind to TLR2 and TLR4 and mediate immune activation in the genital mucosa [31], while endocytosis of HIV activates DCs through TLR7/8 and MyD88 signaling [28, 30]. TLR4 agonists, like lipopolysaccharide (LPS) from gram-negative bacteria, which are translocated into the blood stream during chronic HIV infection has been shown to increase T cell activation [29]. TNF-α and TLR2/1 agonists (PAM3, a lipopeptide from gram-positive bacteria) were shown to increase transmission of HIV in LCs in a human skin explant tissue model [32].

Several approaches have been used to investigate immune responses at the female genital tract, including the use of cervical explant tissues (obtained from elective hysterectomies), cervical cytobrushes and cervicovaginal lavages (CVLs) [33–36]. Cervical explants enable the study of cellular responses within intact mucosal tissues, rather than from single cells, which more closely resembles what happens *in vivo* during HIV infection [33]. This study evaluated the effect of inflammatory cytokines and TLR agonists on DC migration and activation in the female genital tract using human cervical explants, in order to better understand the role of inflammation in promoting HIV infection in the female genital tract. We hypothesized that inflammatory cytokines and TLR agonists would enhance migration and activation of DCs, and this would lead to an increased risk of HIV transmission in the genital tract.

## Methods and Materials

### Participants and sample collection

Seventeen women undergoing elective hysterectomy at Prince Mshiyeni Memorial Hospital in Durban, South Africa, for various medical conditions were enrolled into the study. All laboratory manipulation of hysterectomy tissue was performed at the CAPRISA Mucosal Laboratory, Durban, South Africa. In addition to the cervical tissue collection, cervical swabs were collected for multiplex PCR screening for common sexually transmitted infections (STIs); including Herpes simplex virus (HSV) types 1 and 2, *Neisseria gonorrhoeae*, *Chlamydia trachomatis*, *Treponema pallidum*, and *Trichomonas vaginalis* at the National Health Laboratory Services (NHLS) STI Surveillance Laboratory, as previously described [37]. Women were excluded from this study if their cervical tissue was damaged during surgery, or their tissue did not look healthy for processing by visual inspection. Demographic and clinical patient information was collected using a structured questionnaire. Written informed consent was obtained from all participants. Whole anti-coagulated blood for this study was collected from a separate group of 9 healthy HIV-uninfected female adult participants. The Biomedical Research Ethics Committee (BREC) of the University of KwaZulu-Natal in Durban, South Africa approved the study (Ref: BF:220/12).

### TLR agonists and recombinant cytokines

TLR agonists used in this study included LPS (TLR4 ligand, 100ng/mL; ultrapure, isolated from *Salmonella Minnesota)*; R848, an Imidazoquinoline compound (TLR7/8 ligand, 2000ng/mL); and PAM3 (Pam3CSK_4_ N-palmitoyl-S-[2,3-bis-(palmitoyloxy)-propyl]-(R)-cysteinyl-(lysyl)3-lysine, TLR2/1 ligand, 1000ng/mL) (InvivoGen, France). The recombinant human cytokines used included TNF-α (final concentration 100ng/ml), IL-1β (1000ng/mL), IL-8 (1000ng/mL), MIP-1β (1000ng/mL) (used in a cocktail, with the final concentrations determined by titration) (Biolegend, USA). Concentrations of TLR agonists and cytokines used in these experiments were titrated and optimal doses selected.

### Human cervical explant tissue processing

Cervical tissues were collected and processed as previously described [33]. Cervical tissue was obtained after surgery and immediately placed in transport media containing 10% FBS in RPMI-1640 containing L-glutamine, 1x Penicillin-Streptomycin and 2.5μg/mL fungizone. Tissues were immediately transported to the CAPRISA Mucosal Laboratory for processing, where they were washed in culture media containing 1x non-essential amino acids and 1x Penicillin-Streptomycin in 10% human AB serum in RPMI-1640 containing L-glutamine. The ecto- and endo-cervical regions of fresh tissues were separated and excess tissue discarded. Three of the participants only had samples processed for the endocervix, because the ectocervix was not in good condition, and not all stimulation conditions were included in every experiment due to limitation of the tissue sizes. Ecto- and endo-cervical tissues were trimmed to ~2mm thickness by carefully removing the underlying tissues, and then dissected to ~3mm cubes. For each experiment, cervical explant tissue blocks were washed in culture medium and then incubated in 500μl of media (negative control), cytokine cocktail (containing TNF-α, IL-1β, IL8 and MIP-1β), or stimulated with TLR agonists [LPS (TLR4), PAM3 (TLR2/1), and R848 (TLR7/8)] in a 24-well plate (Corning) in a humidified incubator at 37°C, 5% CO_2_. A non-polarized explant model was used [33]. Two to three cervical explants were incubated per condition. After 24 hours, the medium was collected and replaced with fresh media for another 24 hours after which the medium was again collected and finally the cervical explant tissue was discarded. The medium containing cells that had migrated out of the cervical explants were pooled per condition, washed with PBS twice and stained with a LIVE/DEAD^®^ Fixable Aqua viability dye (Life Technologies, USA) for 10 minutes in the dark. After staining, the cells were washed again twice with 1x PBS and fixed with BD FACS lysing solution. (Becton Dickinson, USA). Fixed cells were frozen down at -80°C until required for staining and analysis by Flow Cytometry. Frozen cells were thawed in batches, washed twice with BD Perm/Wash buffer (BD Biosciences, USA) and stained for 1 hour at room temperature with antibody cocktail containing CD14-Qdot605 (monocyte marker, Invitrogen, USA), CD66-Alexa Fluor 488 (neutrophil marker for exclusion), CD11c-APC/Cy7 (mDCs), CD123-PerCP/Cy5.5 (pDCs), HLA-DR-Alexa Fluor700 (or CCR5-PE), CD1a-PE/Cy7 (Langerhans cells), CD3-Pacific Blue (T cell for exclusion), and CD56-Pacific Blue (NK cells for exclusion) (Biolegend, USA). The cells were washed with Perm/Wash and acquired on the LSRII flow cytometer. At least 50,000 events were acquired from each sample.

**Fig 1:**
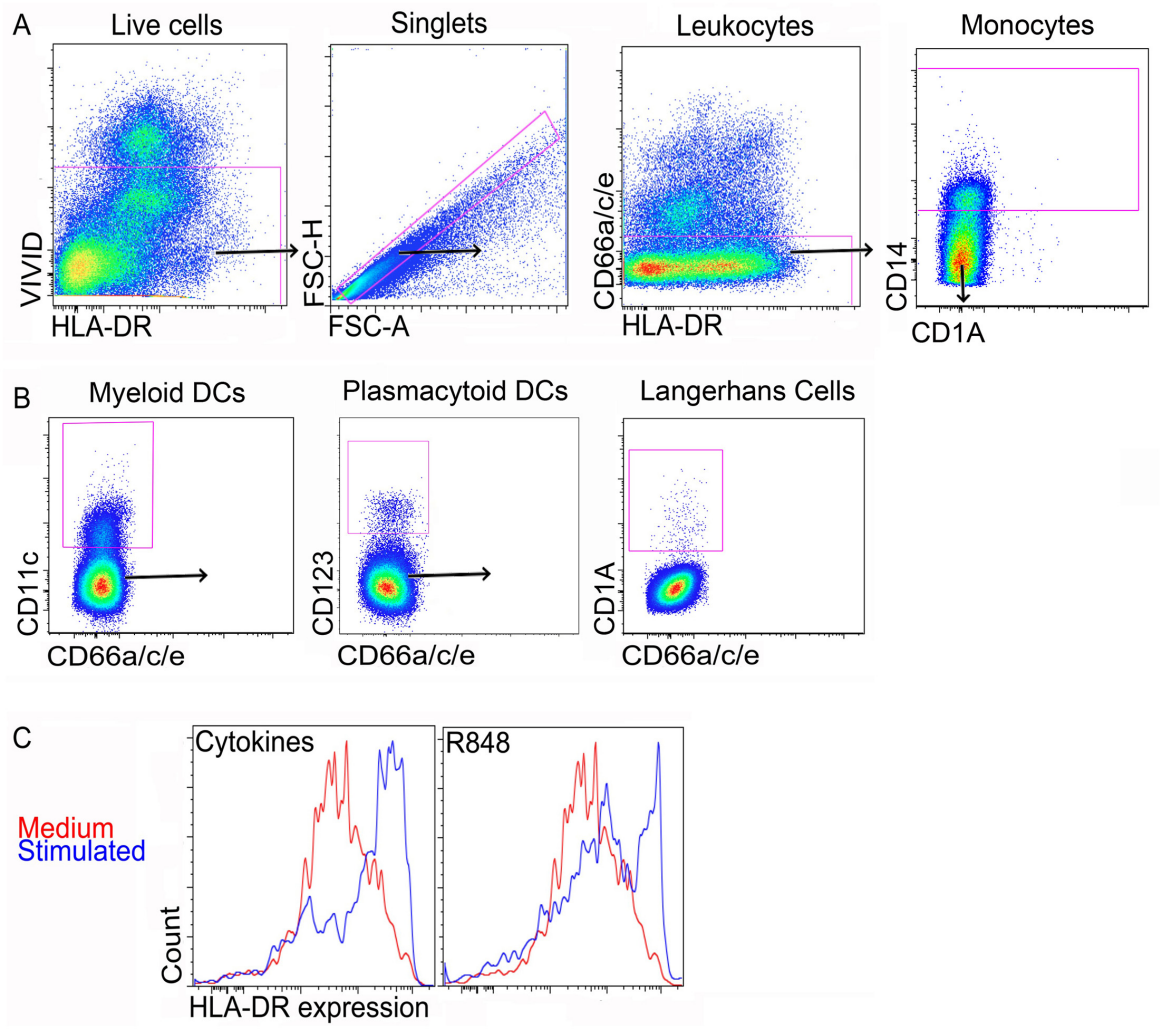
Identification of cervical DC subsets from (ecto) cervical explant cultures. (A) Plots showing the exclusion of dead cells (VIVID^+^), doublets, neutrophils/epithelial cells (CD66a/c/e^+^) and monocytes (CD66a/c/e^−^ CD14^+^). (B) Dendritic cell subsets were identified as mDCs (CD66a/c/e^−^ CD14^−^ CD11c^+^); pDCs (CD66a/c/e^−^ CD14^−^ CD11c^−^ CD123^+^); and LCs (CD66a/c/e^−^ CD14^−^ CD11c^−^ CD123^−^ CD1a^+^); and frequencies of cervical tissue migrating DCs determined, following different stimulation conditions. (C) Histogram showing up-regulation of HLA-DR expression by cervical explant migrating mDCs. Activation of pDCs and LCs was determined in a similar way as mDCs and Median Fluorescent Intensities (MFI) calculated.

DC migration was determined by measuring frequencies of cervical explant creep-out cells in the collected medium. DC activation was determined as the MFI of HLA-DR expression on DC subtypes. DC subtypes were identified after exclusion of doublets, dead cells, T cells (CD3), NK cells (CD56), and neutrophils/epithelial cells (CD66a/c/e). Fig 1 shows the gating strategy used to identify DCs. To normalize for variability between tissues, migration of DCs was expressed as a migration index, calculated as the ratio of stimulated DC frequency over the control DC frequency (medium only). DC activation was expressed as activation index, calculated as the ratio of the MFI of HLA-DR expression by DCs following stimulation over the media only control. HLA-DR was found to be a better marker of DC activation in explants than CD40 expression.

### Whole blood processing

Within 2 hours of collection, 400μl of anti-coagulated whole blood was incubated for 18–20 hours with medium alone (RPMI; negative control), cytokine cocktails (TNF-α, IL-1β, IL-8 and MIP-1β), R848 (TLR7/8 agonist) or a mixture of R848 and cytokines. Following incubation, red blood cells were lysed and white blood cells fixed with BD FACS lysing solution. Fixed white blood cells were frozen in BD FACS lysing buffer at -80°C until staining for flow cytometry. Frozen stimulated cells were thawed and washed twice with BD Perm/Wash buffer (BD Biosciences) and stained for 1 hour at room temperature with antibody cocktail containing CD66a/c/e-PE, CD14-QDot605, CD11c-APC/Cy7, CD123-PerCP/Cy5.5, HLA-DR-Alexa Fluor 700, and CD40-Alexa Fluor 488 (Biolegend). Stained cells were washed and 1 million cells acquired on a BD LSR II flow cytometer.

### Data analysis

Flow cytometry data were analyzed using FlowJo Version 9.6. Results from single-stained and unstained mouse kappa beads were used to calculate compensations. Cell doublets were excluded using forward scatter-area versus forward scatter-height parameters. T cells, NK cells, neutrophils and epithelial cells (for explant samples) were also excluded from the analyses. The MFI for HLA-DR (explants) and CD40 (whole blood) were determined by FlowJo, and samples were excluded if any event numbers for cell populations were less than 20 (for activation only). Any sample with less than 50,000 events during acquisition was also excluded from the analyses. GraphPad Prism Version 6 was used for data presentation and statistical analysis. Comparisons within the same sample were done using a Wilcoxon matched-pairs signed rank test. Comparisons between the different samples were assessed by Mann-Whitney U test, and p values less than 0.05 were considered statistically significant.

## Results

This study modeled the influence of innate TLR signaling and inflammatory cytokines on mucosal DC activation and migration, using non-polarized cervical tissue explants from women undergoing elective hysterectomies. The majority were black South African women (15/17; 88.2%), with a median age of 41 years (IQR 37–47). Of these, 10/17 (58.8%) were HIV-uninfected, and 7/17 (41.2%) were HIV-infected (Table 1). All HIV-infected participants had CD4 cell counts >350 cells/μl. More than half of the women (9/17; 53%) were using hormonal contraception. Clinical reasons for undergoing hysterectomy varied, with 9/17 (53%) women having fibroids, and 8/17 (47%) being treated for high-grade squamous intraepithelial lesion (HSIL). None of the women were positive for other STIs.

**Table 1:** Patient characteristics showing differences between HIV infected and uninfected participants.

Characteristics	HIV+ [n/N (%)]	HIV- [n/N (%)]
N	7/17 (41.1)	10/17 (58.9)
Age [median (IQR); years]	40 (37–41)	46 (38.35–49.5)
Reason for hysterectomy [n/N (%)]		
Fibroids	2/17 (11.8)	7/17 (41.1)
HSIL/CIN III	5/17 (29.4)	3/17 (17.6)
Ethnicity [n/N (%)]		
Black African	7/17 (41.1)	8/17 (47.1)
Indian	0/17 (0)	2/17 (11.8)
Contraception usage [n/N (%)]		
DMPA	5/17 (29.4)	4/17 (23.5)
None	2/17 (11.8)	5/17 (29.4)
n.a	0	1/17 (5.8)

N—Total participants in the study; IQR—Interquartile range; HSIL—High-grade Squamous Intraepithelial Lesion; CIN III—Cervical Intraepithelial neoplasia, Grade 3; DMPA—Depot medroxyprogesterone acetate; n.a—Not available

### DC migration and activation in response to cytokines and TLR agonists

Since we [27], and others [19], have shown that inflammatory cytokines and products of DCs influence mucosal susceptibility to HIV infection, we were interested to model the influence of cytokines and TLR agonists on migration and activation of DCs within human cervical tissues. The cytokine cocktail was chosen based on the observations from our previous study where these cytokines were associated with the risk of HIV acquisition [27].

**Fig 2:**
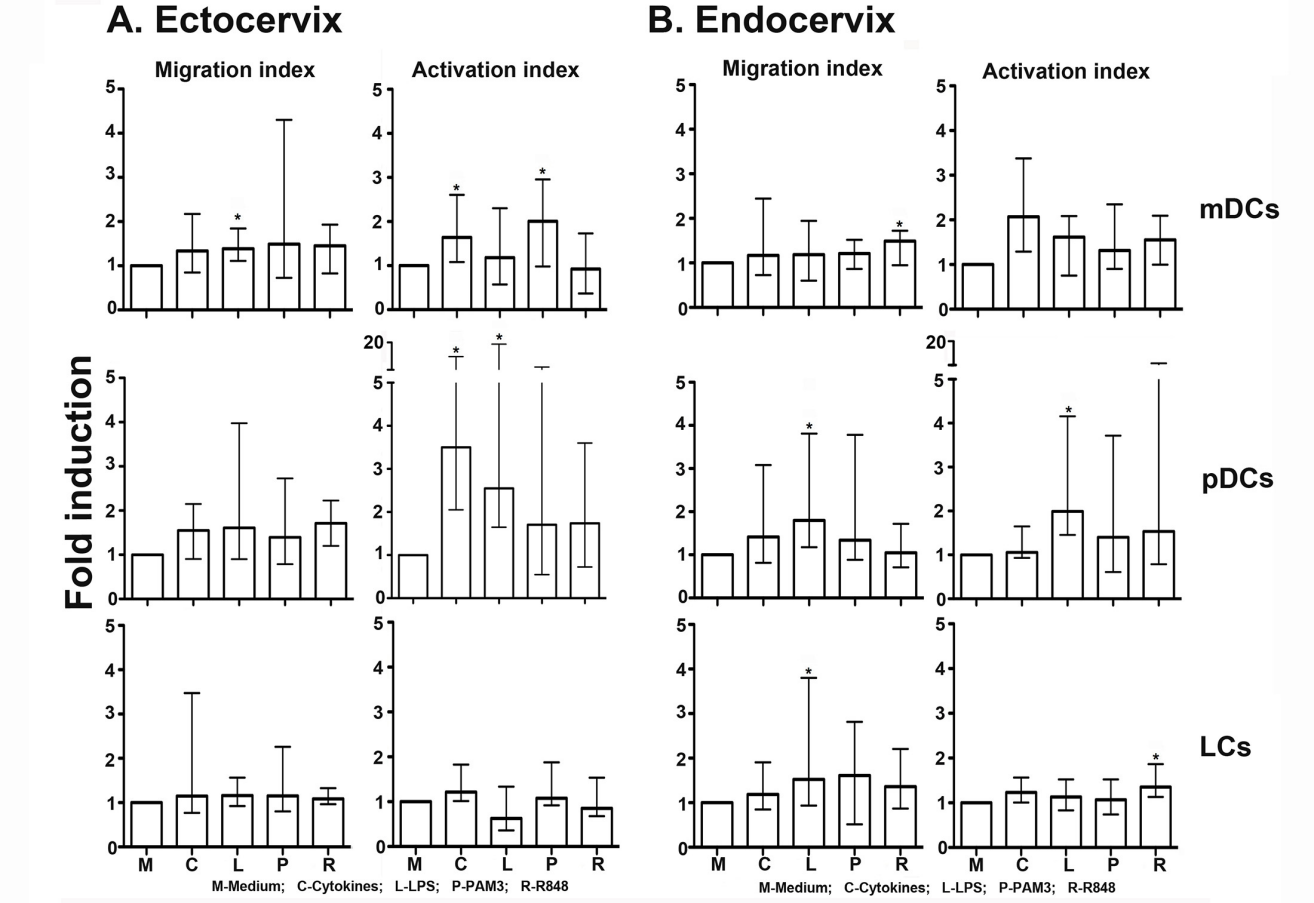
Dendritic cell migration and activation from ectocervical and endocervical tissue explants. Cervical explants were exposed to cytokines (TNF-α, IL-1β, IL-8 and MIP-1β) or TLR agonists (LPS, R848, or PAM3) for 24 hours. Frequencies of DCs that had migrated out of the tissue blocks into collected culture medium, and their activation status was measured in tissue from (A) the ectocervix, and (B) the endocervix. Migration index indicates the ratio of stimulated to unstimulated DC frequencies. Activation index indicates the ratio of stimulated to unstimulated DC HLA-DR MFI. Data is presented as median with interquartile range fold induction or migration above background (medium). Each experiment was performed once and different samples were processed and stimulated for the different conditions: Medium (n = 14), cytokines (n = 14), LPS (n = 14), PAM3 (n = 14) and R848 (n = 14) for ectocervical explants; and Medium (n = 17), cytokines (n = 16), LPS (n = 17), PAM3 (n = 16) and R848 (n = 16) for endocervical explants. Wilcoxon matched-pairs signed rank test was used for statistical analyses. * P-values <0.05.

In ectocervical explants, for all participants regardless of HIV status, samples were processed and stimulated for the different conditions as follows: Medium (n = 14), cytokines (n = 14), LPS (n = 14), PAM3 (n = 14) and R848 (n = 14). Addition of cytokines or TLR agonists induced migration of mDCs (CD66^−^ CD14^−^ CD11c^+^) and pDCs (CD66^−^ CD14^−^ CD11c^−^ CD123^+^), although only migration induced by LPS was significantly higher than that observed in explant cultures with medium alone in mDCs (p = 0.006; Fig 2A). The lack of statistical significance (p = 0.051) in migration of mDCs induced by cytokines may be attributed to a wider variability compared with LPS. Migration of DCs was expressed as a migration index, the ratio of stimulated to unstimulated DC frequencies (S1 Data). In addition, inflammatory cytokines induced activation of migrating mDCs and pDCs, as measured by expression of HLA-DR (p = 0.01 and p = 0.002, respectively). Activation was expressed as activation index, the ratio of HLA-DR MFI of stimulated to unstimulated DCs. TLR agonists also induced activation of migrating mDCs and pDCs [TLR2/1 agonist (PAM3) stimulation of mDCs (p = 0.01) and TLR4 agonist (LPS) stimulation of pDCs (p = 0.005)]. In contrast to these DC subsets, migration and activation of LCs (CD66^−^ CD14^−^ CD11c^−^ CD123^−^ CD1a^+^) from ectocervical explants was similar to culture with media alone. Migration and activation were expressed as ratios to account for possible differences and variation in tissue sizes and responses between individuals. Without normalization, the variation in responses between participants was much greater, though the overall statistics were still similar compared to the normalized data (data not shown).

From endocervical explants, for all participants regardless of HIV status, different samples were processed and stimulated for the different conditions due to limitation of the tissue sizes: Medium (n = 17), cytokines (n = 16), LPS (n = 17), PAM3 (n = 16) and R848 (n = 16). Stimulation with TLR agonists resulted in increased migration of both mDCs and pDCs [Fig 2B; R848 (TLR7/8) on mDCs, p = 0.01; LPS (TLR4) on pDCs, p = 0.002; and LPS on LCs, p = 0.01]. In addition, activation of migrated pDCs and LCs from the endocervical explants was significantly higher following stimulation with LPS (p = 0.001) and R848 (p = 0.005), respectively. Although cytokines and TLR agonists induced further cumulative migration and activation of DCs after 48 hours, differences were not as striking as those observed within the first 24 hours of incubation (data not shown).

We evaluated whether migrating DCs expressed CCR5 (determined in a similar way as HLA-DR expression) in a subset of cervical explants from HIV-uninfected women (n = 4), since CCR5 is an important co-receptor for HIV infection. There was a moderate upregulation of CCR5 expression on mDCs and pDCs following stimulation with LPS compared to medium only (p = 0.071 and 0.065, respectively), although this was not significant (graphs not shown).

### Cervical DCs migrate differently in HIV-infected versus uninfected women

**Fig 3:**
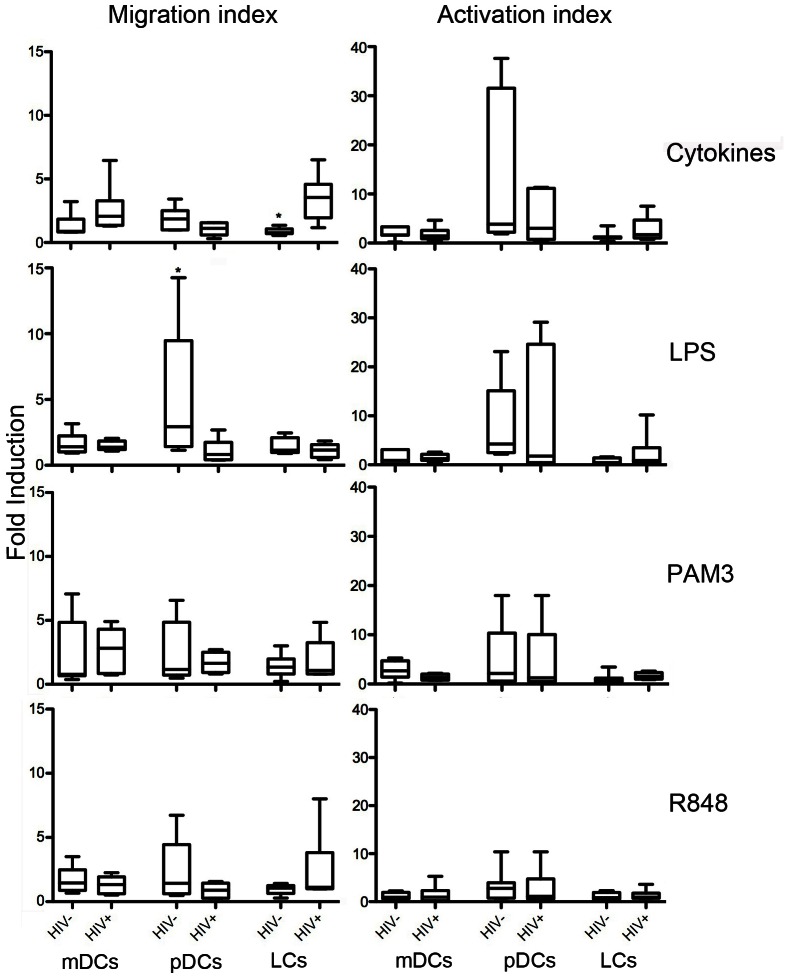
Influence of HIV infection on dendritic cell migration and activation in ectocervical explants. DC migration and activation was compared between HIV-infected (n = 6) and uninfected participants (n = 8). Ectocervical explants from HIV-infected and uninfected participants were stimulated for 24 hours with Medium, cytokines or TLR agonists. Migration and activation of DCs was evaluated and compared between the two groups of participants for the three DC subsets and stimulation conditions. Data presented as Box and Whisker plots of fold migration above background (medium alone). Mann-Whitney test was used for statistical analyses. * P-values <0.05

After evaluating the general effect of inflammation, the effect of inflammation on DC migration and activation in cervical tissues without HIV infection, compared with cervical tissues with HIV infection was investigated. Migration and activation of cervical explant-derived DC subtypes from HIV-infected and uninfected women were compared following stimulation of ectocervical explants with cytokines and TLR agonists (Fig 3), in the same patients as represented in Fig 2 above. Cytokines induced more migration of mDCs and LCs, which was significant for LCs (p = 0.003) in ecto-cervical explant tissue from HIV-infected women than HIV-uninfected women (median [IQR]: 3.539 [1.942–4.577] and 077 [0.702–1.073, respectively]). In contrast, migration of pDCs was increased from cervical explant tissue from HIV-uninfected women, significantly so for LPS (p = 0.01; median [IQR]: 0.833 [0.414–1.738] and 2.932 [1.424–9.467]). While migrating pDCs from HIV-uninfected women tended to have higher levels of activation than HIV-infected women especially for cytokine stimulation and mDCs in response to PAM3, LCs activation tended to be higher in HIV-infected women especially for cytokine and LPS stimulation, though not statistically significant. No statistically significant differences were observed between HIV-infected and uninfected women following endocervical explant stimulation (data not shown).

The differential results observed in both ecto- and endocervical explant stimulation could be influenced by several factors, such as the clinical reason for the elective hysterectomies or use of hormonal contraception (Table 1). In HIV-infected women, HSIL/CIN III were the major reasons for hysterectomy, while fibroids were the main reason for hysterectomies in HIV-uninfected women. There were more HIV-infected women using the long-acting injectable hormonal contraception DMPA (n = 5 vs. n = 4) while more HIV-uninfected women were not using any hormonal contraception (n = 5 vs. n = 2). Unfortunately, the small numbers in the study precluded a more detailed analysis of the effect of these factors on genital DC migration and activation within the HIV negative or positive patients.

We evaluated the general effect of DMPA use on the activation status of migrating cells and found that DMPA use was associated with some changes in activation of genital DCs (S1 Fig). Endocervical explants from DMPA users had increased activation of pDCs in response to cytokines (p = 0.03); and ectocervical explants from DMPA users had increased activation of mDCs only in response to LPS (p = 0.02). Because of the small sample size and moderate differences, the effect of these factors will need to be confirmed in future explant model studies.

### Blood DCs are similarly responsive to cytokine stimulation

As a comparison to tissue, the effect of cytokines on activation and function of DCs from peripheral blood was also evaluated (S2 Fig). Activation of DCs was determined by CD40 expression, with data expressed as MFI of CD40 for stimulated DCs over unstimulated DCs. Inflammatory cytokines induced activation of both mDCs and pDCs in whole blood, with pDCs being more responsive to cytokine stimulation than mDCs. Compared to blood mDCs, blood pDCs were more responsive to cytokine stimulation as pDC from cervical tissues. The responses of whole blood in HIV-infected participants were not evaluated, though it is known that HIV infection alters numbers and responses of blood DCs [38].

## Discussion

The role of pro-inflammatory cytokines and TLR agonists in inducing migration and activation of DCs derived from the female genital tract, using fresh cervical explant tissues, was evaluated in this study and compared to DCs from peripheral whole blood. We showed that cytokines and TLR agonists induce activation of DCs in blood and in the genital tract, and DC migration out of cervical tissue.

TLR agonists modulate function and activation of DCs from peripheral blood [39]. These agonists induce DCs to produce a range of cytokines that are important in immune responses to bacterial and viral infections. Cytokines produced by DCs direct the development of adaptive immune responses, especially the functional polarization of T cells [40]. DC-derived cytokines can also act in an autocrine manner and influence production of cytokines by other DCs [41, 42]. These processes are thought to occur in the female genital tract where DCs and other immune cells are constantly being exposed to various pathogens, anaerobic commensal bacteria and other TLR agonists, resulting from sexual activities or other processes [2, 17]. STIs such as HIV, HSV, chlamydia, gonorrhoea, as well as an altered vaginal flora (bacterial vaginosis) can cause inflammation of the genital tract [27, 43, 44], possibly through stimulation of various TLRs and other PRRs expressed on genital tract cells. Once DCs interact with or detect pathogens, they secrete inflammatory cytokines and this results in the recruitment of more DCs as well as T cells, monocytes/macrophages, neutrophils and NK cells to the genital tract [22, 23, 25, 26]. The absence of STIs among study participants was not unexpected considering that older women generally have less STIs than younger women [45].

We evaluated the role of inflammatory cytokines on the movement of DCs to the female genital tract by evaluating the migration of these cells out of the tissues when cervical explants were treated with a cocktail of inflammatory cytokines (TNF-α, IL-1β, IL-8 and MIP-1β) or activating TLR agonists (PAM3, LPS, and R848). We have shown that these cytokines, present in cervicovaginal lavages, were significantly associated with increased risk for becoming infected with HIV [27]. We found that myeloid DCs and pDCs from cervical explants were more responsive, as assessed through increased migration and activation, to inflammatory cytokines and TLR agonists than LCs. This could be due to differential expression of cytokine receptors and TLRs on these cells [14]. The increase in migration of DCs in response to cytokines and TLR agonists support the hypothesis that pathogenic infections induce migration of DCs within genital tissue, as part of a productive inflammatory response. It has recently been shown that cytokines such as GM-CSF induced enrichment of conventional DCs in the genital tract without causing activation of these cells [46]. Elevated mucosal inflammatory cytokines including TNF-α, IL-1β, IL-8 and MIP-1β have been shown to be associated with increase in recruitment of HIV target cells into the female genital tract, altered expression of mucosal barrier proteins and proteases [47]. In our study, we also found that there was an increase in migration of DCs in response to some stimulation conditions without a corresponding increase in activation. Importantly, we provide evidence from cervical explants that some of the pro-inflammatory cytokines that predicted HIV risk in women [27], and increased CD4^+^ T cell influx to cervical tissues [47], could enhance genital tissue DC migration and activation, possibly suggesting that highly mobile and activated target cells known to transmit HIV are influenced by the inflammatory environment in the female genital tract.

Compared to uninfected women, cervical tissues from HIV-infected women showed altered cell migration and activation, particularly in pDCs. This result was not surprising considering that pDCs preferentially detect and respond to viral infections [21], LCs have been shown to decrease in numbers during chronic HIV infection, but it is unclear if their activation profile is altered [48]. Given that LCs and pDCs express low levels of TLR4 [49], the response to LPS in this study may be attributed to by-stander activation. While inflammation is needed to curb pathogenic genital infections, the production of cytokines and the recruitment of DCs and T cells to the genital mucosa inadvertently serve to fuel HIV transmission as HIV binds receptors expressed on these activated cells to promote its propagation [17, 19, 26, 27, 50].

One of the strengths of this study was our ability to evaluate the effects of female genital tract inflammation on migration and activation of DCs that are the initial primary targets of HIV infection, in a whole tissue model. The findings observed in cervical explant cultures from the female genital tract were also confirmed in whole blood. A limitation of this study is that we were unable to differentiate active migration of DCs in this *in vitro* human tissue model from passive diffusion of cells, which might have contributed to accumulation of cells outside of the tissue. Although we confirmed that viability of cells assessed by flow cytometry did not differ significantly between stimulation conditions, we did not include additional markers of tissue damage in the explant tissues. Furthermore, we focused only on the effect of cytokines only on DCs and not on other HIV target cells such as CD4^+^ T cells. This will be addressed in future studies, which will also include analyses of the activation state of cells within the tissues using immunohistochemistry or other relevant assays before and after stimulation. Another limitation was a relatively heterogeneous study population of older women with genital tract pathology requiring elective hysterectomies at the time of study. The possibility that the cervical tissue may already have been in an inflamed state at the time of collection or inflammation induced during processing could have influenced the level of activation achieved following stimulation with cytokines or TLR agonists, and the lack of correlation between migration and activation. Furthermore, some women were HIV positive, with unknown immune status, which could have altered the immune response. In addition, there was no appropriate one-size-fits-all positive control ligand for the cervical tissues: LPS is generally used as positive control to stimulate myeloid cells and was used in this study to activate mDCs, while R848 was used to activate both mDCs and pDCs, due to differential TLR expression on these cells [2, 49]. Although these agonists induced high levels of activation and cytokine expression by mDCs and pDCs in whole blood, responses from cervical tissue was more moderate, possibly reflecting the *ex vivo* inflamed state of surgical tissue as a likely confounder. The small sample size precluded adequate analyses of the effect of DMPA use on genital tract-derived DC activation.

In conclusion, this study elucidated the role of inflammatory cytokines in DC migration and activation in the female genital tract. DCs are targets in HIV infection and their role in transmission is critical as they can either be infected or promote transmission by transferring HIV to T cells found in the genital sub-mucosa. This migration and activation of DCs could enhance HIV transmission in the genital tract. Further studies are underway to evaluate the role of inflammation and cell migration/activation in altering the effectiveness of microbicides to prevent HIV transmission in the female genital tract.

## Supporting Information

S1 Data Data file for the results reported in the manuscript. Data set for frequencies of migrating cells and median fluorescent intensities of HLA-DR expression on each cell subset, migration and activation indices in the ectocervix and endocervix for each stimulation condition, HIV status and the use of hormonal contraception for each participant.
(XLSX)

S1 Fig Association between use of hormonal contraception and activation of migrating genital tract-derived DCs. The effect of DMPA use on activation of genital tract DCs was evaluated after stimulation with cytokines, LPS, PAM3 and R848. The activation of DCs was compared between patients using DMPA (DMPA+) and those not using DMPA (DMPA-). The fold induction over background (activation index) was determined for both ectocervix (left panel) and endocervix (right panel). Data presented as Box and Whisker plots. Mann-Whitney test was used for statistical analyses * P-values < 0.05.
(TIFF)

S2 Fig Influence of pro-inflammatory cytokines on blood dendritic cell activation. Activation of whole blood mDCs and pDCs from 9 participants was measured after 18 hours stimulation with pro-inflammatory cytokine cocktails (TNF-α, IL-1β, IL-8 and MIP-1β). Red blood cells were lysed and white blood cells stored at -80°C and later thawed and stained with fluorescent-conjugated antibodies against markers for DC phenotype and activation. Wilcoxon matched-pairs signed rank test was used for statistical analyses. P values <0.05 were considered significant.
(TIFF)
