# Supplementary figures and images for: Modulation of Female Genital Tract-Derived Dendritic Cell Migration and Activation in Response to Inflammatory Cytokines and Toll-Like Receptor Agonists
Source: PLoS One. 2016 May 12;11(5):e0155668. doi: 10.1371/journal.pone.0155668 (PMC4865202; doi:10.1371/journal.pone.0155668)

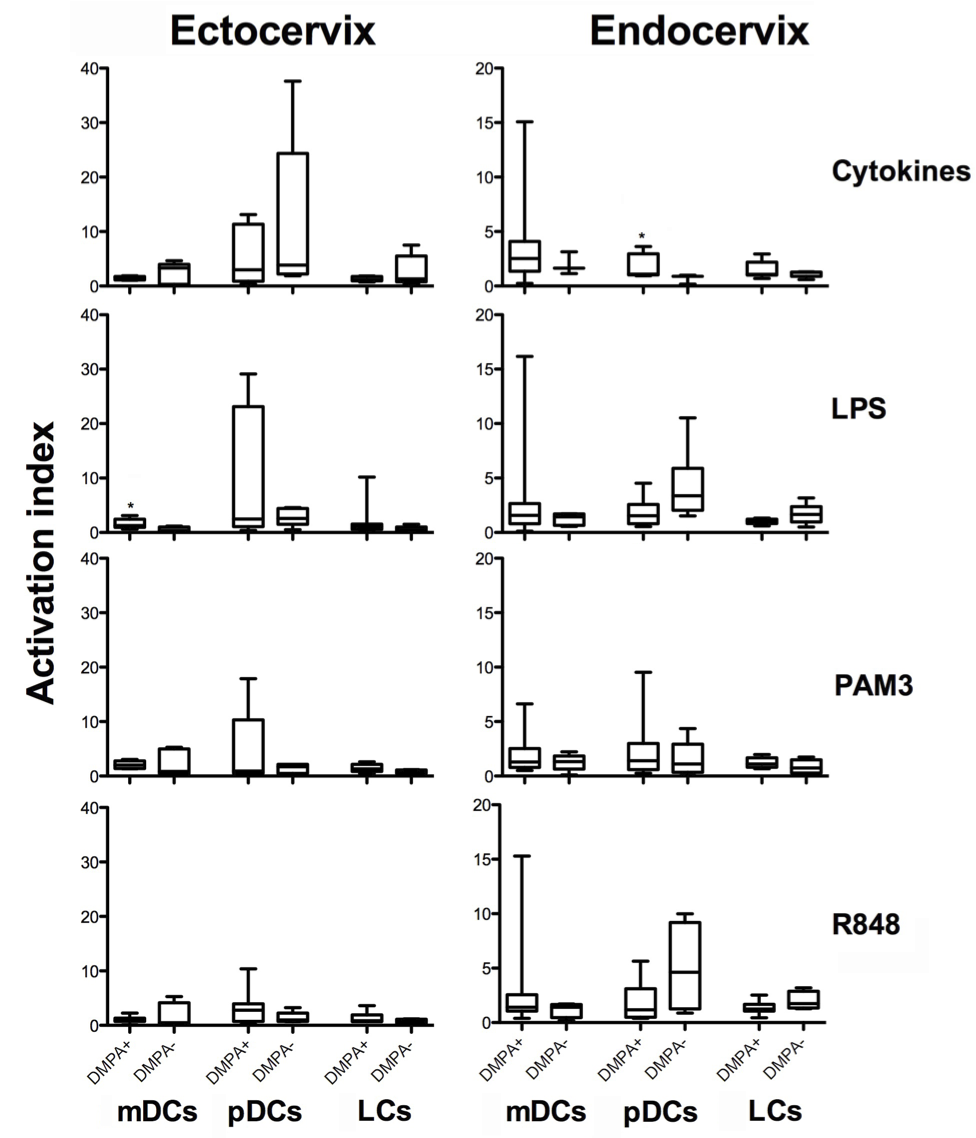

Supplement: S1 Fig — The effect of DMPA use on activation of genital tract DCs was evaluated after stimulation with cytokines, LPS, PAM3 and R848. The activation of DCs was compared between patients using DMPA (DMPA+) and those not using DMPA (DMPA-). The fold induction over background (activation index) was determined for both ectocervix (left panel) and endocervix (right panel). Data presented as Box and Whisker plots. Mann-Whitney test was used for statistical analyses * P-values < 0.05. (TIFF) [file pone.0155668.s002.tiff]

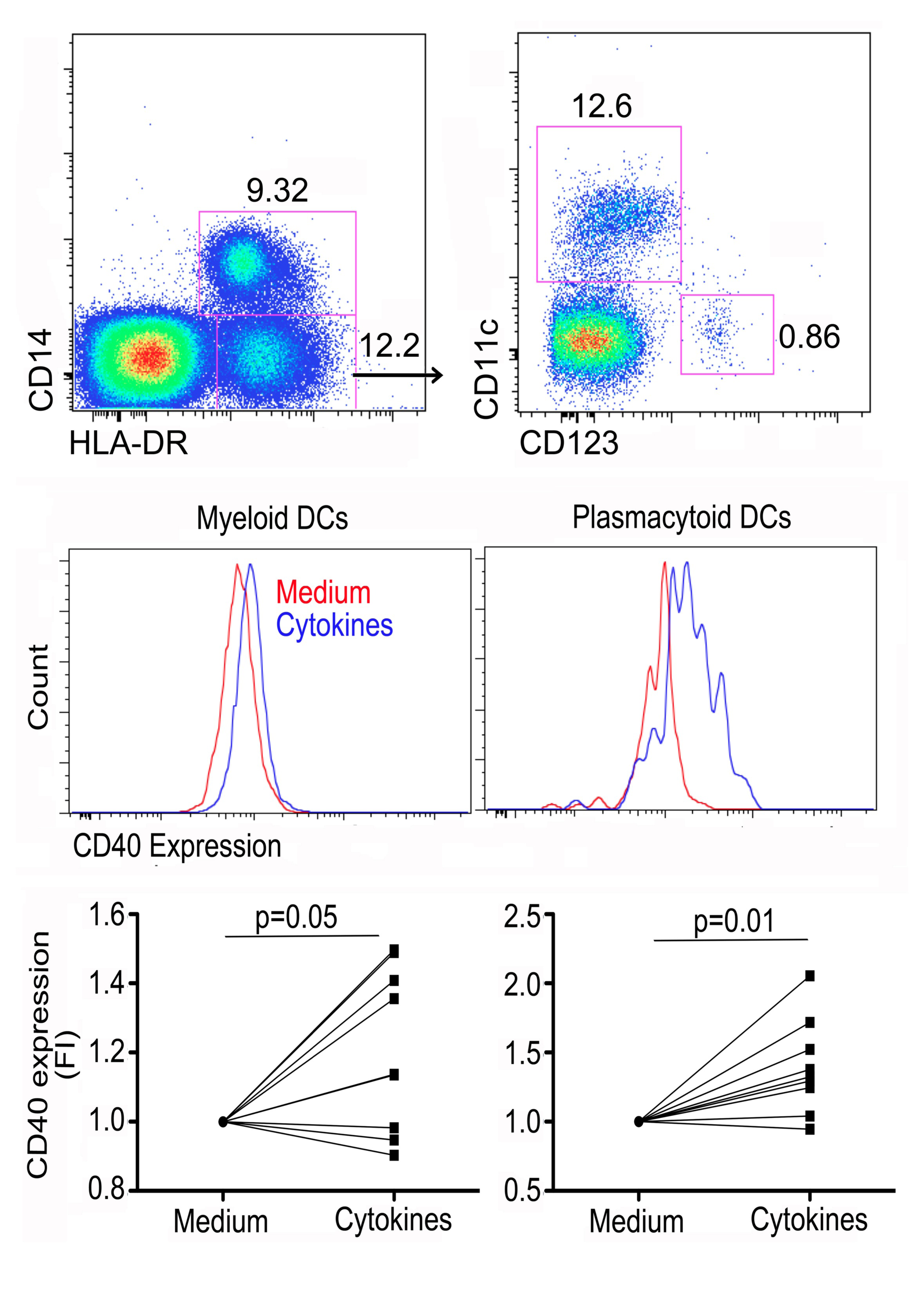

Supplement: S2 Fig — Activation of whole blood mDCs and pDCs from 9 participants was measured after 18 hours stimulation with pro-inflammatory cytokine cocktails (TNF-α, IL-1β, IL-8 and MIP-1β). Red blood cells were lysed and white blood cells stored at -80°C and later thawed and stained with fluorescent-conjugated antibodies against markers for DC phenotype and activation. Wilcoxon matched-pairs signed rank test was used for statistical analyses. P values <0.05 were considered significant. (TIFF) [file pone.0155668.s003.tiff]
